# ECT associated musical hallucinations in an elderly patient: a case report

**DOI:** 10.1186/1744-859X-5-10

**Published:** 2006-08-04

**Authors:** Raguraman Janakiraman, Keith Wildgoose, Kalyan Seelam

**Affiliations:** 1 Department of Psychiatry, Sheffield Care Trust, S5 7JT, UK; 2 Department of Psychiatry, Doncaster Royal Infirmary, DN2 5LT, UK

## Abstract

Electro Convulsive Therapy (ECT) is a medical treatment for severe mental illness in which small, carefully controlled electricity is applied to the brain. This electric stimulation is done in conjunction with anesthesia and muscle relaxant medications to produce a mild generalized seizure. This is used to treat a variety of psychiatric disorders. This is most effective in the treatment of severe depression providing a rapid relief. We report and discuss an unusual presentation of a ninety three year old lady with a diagnosis of Major Depressive Disorder, Recurrent, Severe with Psychotic features (296.34) who experienced musical hallucinations whilst she was treated on ECT. Clinically there was an inverse relationship between the biological symptoms of depression and musical hallucination during the ECT management. Though similar reports have never been reported earlier, we noticed a good association between the initiation of ECT and musical hallucination in our patient. The patient stopped experiencing musical hallucinations and improved of her biological symptoms of depression completely after the full course of ECT.

## Background

Hallucinations may occur in any sensory modalities. Formed musical hallucinations (i.e. Perception of either vocal or instrumental melodies) reported in English literature to date have typically been associated with marked hearing loss, advanced age, female sex (71%), lack of response to treatment and associated psychopathology [1] but has been concluded that hearing loss is neither a necessary nor sufficient condition for the occurrence of musical hallucinations [1]. Tumor like Astrocytoma, Temporal lobe epilepsy, raised intra cranial tension, drug induced (tricyclics, Propronolol etc ;), meningitis and psychotic illness were shown to cause palinacousis and musical hallucinations [2,3].

Musical hallucinations are pseudo hallucinations that originate in memory representations and they may undergo a transition to true hallucination. In musical hallucination spatial projection is less definite. Sometimes they are perceived like the tinnitus in patient's own head. The quality is usually very intense and distinct often very loud. They vary from elementary sounds to instrumental music, vocal music, bird songs, bells, pieces of melodies or sentences, religious music etc:- We report an unusual presentation of an elderly lady who had experienced musical hallucinations whilst being treated with Electro Convulsive Therapy. Though this has never been reported earlier, there seemed to have a good association between the initiation of Electro convulsive therapy and musical hallucination in our patient.

## Case presentation

We report a case of 93 year old British lady, moderately built, living in a residential home. She was admitted for a few times in the past with a diagnosis of Major Depressive episode, Severe with Psychotic symptoms. She had been treated and stabilized with Mirtazapine 30 mg per day. She never smoked or consumed alcohol. She had no past history of chronic obstructive pulmonary disease.

Recently, she was admitted to Functional Ward with a diagnosis of Major Depressive Disorder, Recurrent, Severe with Psychotic features (296.34). She presented to us with poor sleep, anergia, easy fatigability, poor concentration and low mood. She was noted to have psychomotor retardation with tear laden eyes and Veraguth's folds. She firmly believed that dead people were around her and trying to harm her. She also admitted hearing voices of dead people telling her to die. She appeared distressed and refused to accept food, drink and medications. She was apparently stable in her mood until a month prior to the current admission. She contracted lower respiratory tract infection a month ago, for which she was successfully treated with antibiotics. Despite this, her general condition deteriorated and she refused her medications, food and drink.

Her general physical condition deteriorated further due to poor hydration and self neglect. On admission, her neurological examination was unremarkable. Her CT Brain showed age related generalized atrophic changes. There was no evidence of space occupying lesion, focal areas of hemorrhage or infarction in the brain. Since there was no history of seizures, focal neurological deficits and CT Brain scan showing age related changes, EEG was not performed.

It was felt in Multidisciplinary team that she would improve with a short term course of eight ECTs. As she was unable to consent, second opinion was sought and she was posted for bipolar ECT twice a week for four weeks. Pre-ECT investigations, ECG, Chest X Ray, Blood sugar were normal. The ECT was carried out in conjunction with anesthesia and muscle relaxant medications to produce a mild generalized seizure. Though urea and creatinine levels were deranged initially, they got stabilized once the hydration had improved.

After the first two ECTs, she started accepting her food, drink and medications. Her general condition and hydration improved. She was cheerful and humorous. She started maintaining a good rapport, with appropriate conversation. She was more optimistic about her future.

Couple of days after the second ECT, she complained of hearing musical notes and songs during most of the times in a day. She heard "Rose in a garden of weeds" which she pointed out to be her favorite song during her childhood. This musical hallucination persisted up to few days after the last ECT. Though she experienced the musical hallucinations, they were not distressing to her. The patient neither experienced the hallucinations nor could recall the song after the completion of the course of ECT. Her depressive symptoms improved. She was stabilized on Mirtazapine 30 mg per day again and discharged to the residential home.

Musical hallucinations are not uncommon in many psychiatric illnesses, dementias, organic causes, drug induced problems etc; none has been reported to be associated with ECT therapy. Though Wengel SP et al reports that ECT is very effective in treating depression and musical hallucinations [1], in our patient; ECT which was started for severe depression has shown a temporal association with the origin of musical hallucination during recovery from depression. Hearing loss is neither a necessary nor sufficient condition for the occurrence of musical hallucinations.

## Conclusion

Palinacousis (Auditory perseveration) is a rarely reported symptom of temporal lobe dysfunction. Patterson MC et al, reports palinacousis in a patient suffering from grade IV astrocytoma over left temporal region with generalized seizure. The patient's palinacousis improved with antiepileptic therapy [2]. Neuroanatomically patients with musical hallucination in comparison with controls had increased regional cerebral blood flow in left temporal region and left angular gyrus [4]. Takeshi Terao et al reports similarities between palinacousis and musical hallucinations, though there are some differences in their contents. Both the symptoms are often associated with seizure activity and there have been several case reports where anticonvulsants were successfully used to treat both symptoms [5]. Another case report by Roberts DL et al has shown episodic musical hallucinations associated with seizures that led to the discovery of 2 small intracranial aneurysms [6]. These findings indicate the possibility that there might be a common pathway generating musical hallucinations and palinacousis to explain the cause and effect relationship with seizure activity.

More over in the case reported by us, patient developed the musical hallucination immediately after second ECT which persisted during the complete course and disappeared few days after the last ECT. She neither experienced the hallucination nor could remember the song she heard which could possibly be because of ECT induced memory loss. The pathophysiology of musical hallucination is discussed considering the theories of deafferentiation including the concept of auditory Charles-Bonnet Syndrome of sensory auditory deprivation, of parasitic memory, spontaneous activity of cognitive network module. So, musical hallucinations are a phenomenon with heterogeneous clinical and pathophysiological backgrounds of other mental disorders [7]. The pathogenetic role of the musical hallucination is mostly interpreted in terms of perceptual release theory [8]. The continuous input of sensory stimuli is thought to inhibit the memory traces stored in neuronal circuits, always ready for reactivation. If this input is reduced below threshold level with sufficient cortical arousal, this results in disinhibition, release and conscious perception of engrams.

In a phenomenological study of thirty consecutive referrals of old people with musical hallucination concentrating on the names of the melodies heard, Hymns and Christmas carols was the most common experience with "Abide with me" particularly being the most frequent [9].

In this report, interestingly musical hallucinations seem to have good association with the course of ECT which is not reported so far. Further neuroanatomical study in future may unravel the complex issues behind this association. This study involves only a single individual and therefore may not be representative of the general group. In future, this needs further exploration by systematically analyzing a large sample with control group.

## Competing interests

The author(s) declare that they have no competing interests.

## Authors' contributions

**RJ **made substantial contributions to conception and design of the case report, drafted the manuscript and revised it critically for important intellectual content. **KW **initiated to write up the manuscript and had given the final approval of the version to be published. **KS **was involved in drafting and revising the case report along with first author.
